# Apremilast, a novel phosphodiesterase 4 (PDE4) inhibitor, regulates inflammation through multiple cAMP downstream effectors

**DOI:** 10.1186/s13075-015-0771-6

**Published:** 2015-09-15

**Authors:** Miguel Perez-Aso, M. Carmen Montesinos, Aránzazu Mediero, Tuere Wilder, Peter H. Schafer, Bruce Cronstein

**Affiliations:** Department of Medicine, New York University School of Medicine, 550 First Ave., New York, NY 10016 USA; Departament de Farmacologia, Facultat de Farmàcia, Universitat de València, 46100 Burjassot, Spain; Department of Translational Development, Celgene Corporation, Summit, NJ USA; Division of Translational Medicine, Department of Medicine, New York University School of Medicine, 550 First Avenue, MSB251, New York, NY 10016 USA

## Abstract

**Introduction:**

This work was undertaken to delineate intracellular signaling pathways for the PDE4 inhibitor apremilast and to examine interactions between apremilast, methotrexate and adenosine A_2A_ receptors (A_2A_R).

**Methods:**

After apremilast and LPS incubation, intracellular cAMP, TNF-α, IL-10, IL-6 and IL-1α were measured in the Raw264.7 monocytic murine cell line. PKA, Epac1/2 (signaling intermediates for cAMP) and A_2A_R knockdowns were performed by shRNA transfection and interactions with A2AR and A2BR, as well as with methotrexate were tested *in vitro* and in the murine air pouch model. Statistical differences were determined using one or two-way ANOVA or Student’s t test. The alpha nominal level was set at 0.05 in all cases. A P value of < 0.05 was considered significant.

**Results:**

*In vitro*, apremilast increased intracellular cAMP and inhibited TNF-α release (IC_50_=104nM) and the specific A_2A_R-agonist CGS21680 (1μM) increased apremilast potency (IC_50_=25nM). In this cell line, apremilast increased IL-10 production. PKA, Epac1 and Epac2 knockdowns prevented TNF-α inhibition and IL-10 stimulation by apremilast. In the murine air pouch model, both apremilast and MTX significantly inhibited leukocyte infiltration, while apremilast, but not MTX, significantly inhibited TNF-α release. The addition of MTX (1 mg/kg) to apremilast (5 mg/kg) yielded no more inhibition of leukocyte infiltration or TNF-α release than with apremilast alone.

**Conclusions:**

The immunoregulatory effects of apremilast appear to be mediated by cAMP through the downstream effectors PKA, Epac1, and Epac2. A2AR agonism potentiated TNF-α inhibition by apremilast, consistent with the cAMP-elevating effects of that receptor. Because the A2AR is also involved in the anti-inflammatory effects of MTX, the mechanism of action of both drugs involves cAMP-dependent pathways and is therefore partially overlapping in nature.

**Electronic supplementary material:**

The online version of this article (doi:10.1186/s13075-015-0771-6) contains supplementary material, which is available to authorized users.

## Introduction

Accumulating data since the 1950s describe the properties of cyclic adenosine monophosphate (cAMP) as a pivotal second messenger in the regulation of inflammatory responses [1]. Cyclic AMP-specific phosphodiesterases (PDE), therefore, have emerged as a new target for the treatment of numerous inflammatory conditions (reviewed in [2]) as the anti-inflammatory properties of PDE inhibitors such as theophylline were being demonstrated during the 1970s [3]. cAMP-specific phosphiodiesterase type 4 (PDE4), a critical regulator of intracellular cAMP levels and compartmentalization [4], is mainly expressed within inflammatory cells [5]. Thus, inhibition of PDE4 suppresses the expression of TNF-α, among other cytokines and chemokines, by T cells and monocytes [6–8]. PDE4 inhibitors are well-characterized pharmaceutical agents with a broad range of anti-inflammatory activity, as documented by numerous studies of low molecular-weight PDE4 inhibitors, such as rolipram [9].

The clinical development of a class of PDE4 inhibitor anti-inflammatory agents has been hindered by their side effects, principally nausea and emesis [10]. Indeed, despite their potential as anti-inflammatory agents, PDE4 inhibitors have failed clinical trials due to the high prevalence of these and other side effects [11] such as headache, diarrhea, fatigue, dyspepsia, nasopharyngitis and gastroenteritis [12]. Thus, a major pharmaceutical research focus in the field of chronic inflammatory diseases was the development of novel PDE4 inhibitors with high therapeutic index [13]. The novel PDE4 inhibitor, apremilast, has a higher therapeutic index and was developed, introduced to the clinic [14, 15] and recently approved in the USA for the treatment of psoriatic arthritis (reviewed in [2]).

Unlike TNF-α inhibitors, which bind directly to TNF-α, PDE4 inhibitors inhibit TNF-α production at the level of gene expression and do not completely suppress TNF-α levels in clinical settings. Rather, apremilast causes a broad, but not complete, inhibition of multiple pro-inflammatory mediators [16]. It is well-known that PDE4 inhibition promotes intracellular accumulation of cAMP, activation of Protein kinase A (PKA) and phosphorylation of the cAMP-response element binding protein (CREB), which, in turn, suppresses the transcription of numerous cytokines, such as TNF-α, exerting therefore an overall anti-inflammatory effect [9]. However, the specific impact of apremilast on the expression of different cytokines and the role of other downstream effectors of cAMP, i.e., Exchange protein directly activated by cAMP (Epac)1 and Epac2, in the mechanism of action of apremilast have not been previously addressed, nor has the interaction of apremilast with other systems that regulate cAMP levels, such as adenosine receptors, in particular with the A_2A_ adenosine receptor (A_2A_R), which mediates many of the anti-inflammatory actions of methotrexate (MTX), the cornerstone treatment for rheumatoid arthritis [17]. Therefore, the aim of the present work was to analyze the downstream pathways triggered by apremilast and to test potential interactions of apremilast with MTX, and with the adenosine receptors in both in vitro and in vivo models of inflammation.

## Materials and methods

### Air pouch model

As previously described [17], male mice were given weekly intraperitoneal injections of either MTX (1mg/Kg) or vehicle (phosphate-buffered saline; PBS) for 4 weeks. Air pouches were generated by subcutaneous injection of 3 ml of sterile air and reinflated with 1.5 ml of sterile air 2 days later. Vehicle (0.5 % carboxymethylcellulose and 0.25 % Tween 80) or apremilast (5 mg/Kg) were orally dosed, with a syringe through a blunt-ended curved feeding tube, 24 h and 1 h before inflammation was induced on day 6 by injection of 1 ml of 2 % carrageenan suspension. Four hours later, mice were killed by CO_2_ narcosis, and exudates harvested with 2 ml PBS. Leukocytes were counted in a hemocytometer chamber and concentrations of cytokines were measured by ELISA or by the Luminex platform as described below. All protocols followed internationally recognized guidelines and were approved by the New York University School of Medicine Institutional Animal Care and Use Committee (NYU SoM, IACUC, protocol number 130412).

### Histology

Four-micron formalin-fixed, paraffin-embedded tissue sections were stained with H&E or with toluidine blue histochemicals by incubating sections in aqueous 0.01 % toluidine blue for 5 minutes. Slides were washed in distilled water, quickly dehydrated through graded alcohols, cleared in xylene and mounted with synthetic permanent media [18].

Immunohistochemistry was performed on four-micron formalin-fixed, paraffin-embedded mouse skin tissue sections using rabbit anti-mouse CD3 (Ventana Medical Systems Tucson, AZ, USA) clone 2GV6, rat anti-mouse CD45R (BD Biosciences San Diego, CA, USA) clone RA3-6B2, rat anti-mouse CD68 (Abd Serotech, Raleigh, NC, USA) clone FA-11 and rat anti-mouse neutrophil (Abcam Cambridge, MA, USA) clone NIMP-R14. In brief, sections were deparaffinized in xylene (three changes), rehydrated through graded alcohols (three changes 100 % ethanol, three changes 95 % ethanol) and rinsed in distilled water. For CD3, heat-induced epitope retrieval was performed in a 1200-Watt microwave oven at 100 % power in 10 mM sodium citrate buffer, pH 6.0 for 20 minutes. Sections were allowed to cool for 30 minutes and then rinsed in distilled water. Antibody incubations and detection were carried out at 37 °C on a Discovery or NEXes instrument (Ventana Medical Systems Tucson, AZ, USA) platform using Ventana reagent buffer and detection kits unless otherwise noted. Endogenous peroxidase activity was blocked with hydrogen peroxide. Tissue sections for CD68 and anti-neutrophil were digested at 37 °C with alkaline endopeptidase for 12 and 6 minutes, respectively. All antibodies were diluted in Dulbecco’s Phosphate-Buffered Saline (Life Technologies Grand Island, NY, USA). CD3 and CD45R were diluted 1:10 and incubated for 30 minutes. CD68 was diluted 1:10 and anti-neutrophil 1:1600 and incubated for 12 h at room temperature. CD3 was detected with biotinylated goat anti-rabbit (Vector Laboratories Burlingame, CA USA) diluted 1:200. CD45R and anti-neutrophil were detected with biotinylated goat anti-rat diluted 1:200 and CD68 was detected using biotinylated rabbit anti-rat, mouse absorbed (Vector Laboratories Burlingame, CA, USA) diluted 1:100. Secondary antibodies were incubated for 30 minutes at 37 °C. This was followed by the application of streptavidin-horseradish-peroxidase conjugate. The complex was visualized with 3,3 diaminobenzidene and enhanced with copper sulfate. Slides were washed in distilled water, counterstained with hematoxylin, dehydrated through graded alcohols, cleared in xylene and mounted with synthetic permanent media. Appropriate positive and negative controls were included with the study sections.

### cAMP measurements

Intracellular cAMP was measured with the direct cAMP ELISA kit from Enzo Life sciences (Plymouth Meeting, PA, USA). Fifty-percent-confluent Raw 264.7 cells were starved for 24 h and stimulated at the indicated concentrations of apremilast for 30 minutes, and then with lipopolysaccharide (LPS) for 20 minutes, and cAMP was analyzed according to the manufacturer’s protocol.

### TNF-α measurement

Raw 264.7 cells (100,000) were grown in 96-well plates. After 24 h, cells were stimulated with vehicle (final concentration of 0.025 % dimethyl sulfoxide (DMSO)) or with apremilast at the indicated concentrations. After 30 minutes cells were stimulated with LPS (L5886; Sigma, St Louis, MO, USA) 1 μg/ml for 4 h. When studying CGS21680 (1063; Tocris Bioscience, Ellisville, MO, USA), SCH58261 (2270; Tocris Bioscience), ZM241385 (1036; Tocris Bioscience), BAY60-6583 (4472; Tocris Bioscience), or GS6201 (4727; Tocris Bioscience), the adenosine receptor ligands were added 15 minutes before apremilast. Methotrexate (Hospira Inc, Lake Forest, IL, USA) was added 24 h and 1 h before apremilast. Supernates were then collected and TNF-α levels were quantified with the Mouse TNF-α Quantikine ELISA Kit (MTA00B; R&D systems; Minneapolis, MN, USA) following the manufacturer’s instructions.

### IC50 calculation and statistics

IC50 (EC50) calculations were made using non-linear regression, sigmoidal dose–response, constraining the top to 100 % and bottom to 0 %, allowing variable slope, using GraphPad Prism v6.00.

### Western blotting

Seventy-percent-confluent Raw 264.7 cells were starved for 24 h and stimulated with apremilast for 30 minutes and then with LPS for different time points (n = 4), Cells were lysed with radioimmunoprecipitation assay (RIPA) buffer and protein concentration was determined by bicinchoninic acid (BCA). Protein (4 μg) was subjected to 7.5 or 10.0 % SDS-PAGE and transferred to a nitrocellulose membrane. Nonspecific binding was blocked with TBS/Tween-20 0.05−3 % BSA. Membranes where incubated overnight (4 °C) with primary rabbit polyclonal anti-pCREB (Abcam, Cambridge, MA, USA), mouse monoclonal anti-CREB (Abcam), rabbit polyclonal anti-PDE4 (Abcam) and mouse monoclonal anti-Actin (1:1000 each). Membranes were incubated with goat anti-rabbit IRDye 800CW 1:10000 and goat anti-mouse IRDye 680 RD 1:10000 (Li-cor Biosciences) in the dark. Proteins were visualized by Li-cor Odyssey equipment, which detects near-infrared fluorescence. As each secondary antibody emits a signal in a different spectrum, reprobing with actin (to check that all lanes were loaded with the same amount of protein) was performed simultaneously with primary antibody incubation. Intensities of the respective band were quantitated by densitometric analysis using Image Studio 2.0.38 software (Li-cor Biosciences). Variations in band intensity were expressed as the percent of unstimulated controls, to minimize disparities among different experiments.

### Quantitative reverse transcription (RT)-PCR

Total RNA was extracted and purified using the RNeasy Mini Kit (QIAGEN, Valencia, CA, USA) according to the manufacturer’s protocol. Relative quantification of gene expression was performed using real-time RT-PCR on the Mx3005P Real-Time PCR System (Strategene, Agilent technologies Santa Clara, CA, USA) with SYBR Green (Agilent technologies, 600548, Santa Clara, CA, USA) according to the manufacturer’s protocol. The following primers were used in real-time PCR amplification: Glyceraldehyde-3-phosphate dehydrogenase (GAPDH) forward: 5 -CTACACTGAGGACCAGGTTGTCT −3, reverse: 5- GGTCTGGGATGGAAATTGTG −3; Protein kinase A (PKA) forward: 5- CAGGAAAGCGCTCCAGATAC −3, reverse: 5- AAGGGAAGGTTGGCGTTACT −3; Epac1 forward: 5- GTTGTCGACCCACAGGAAGT −3, reverse: 5- ACCCAGTACTGCAGCTCGTT −3; Epac2 forward: 5- GCATTGAGCAGGAGGACTTC −3, reverse: 5- AACGTGGGGTTCAATGAGAG −3; A_2A_R forward: 5- AGCCAGGGGTTACATCTGTG −3, reverse: 5- TACAGACAGCCTCGACATGTG −3. mRNA abundance was determined relative to that of GAPDH.

### Luminex assay

Quantification of cytokines and chemokines was performed using Luminex x-MAP technology (Luminex Corp, Austen TX, USA). Tissue culture supernatants and mouse exudates were analyzed for expression of IL-1α, IL-6 and IL-10 using a Milliplex multi-analyte magnetic bead panel from EMD Millipore (MCYTOMAG-70K, Billerica, MA, USA). Assays were performed according to the kit protocol using the appropriate matrix solution (culture media or PBS for supernatants and exudates, respectively). Data were collected on a Luminex 200 instrument and analyzed using Analyst 5.1 software (Millipore) with four-parameter logistic curve fitting. Samples were assayed in duplicate. All standard curves generated from the known reference cytokine concentrations supplied by the manufacturer had *R*^2^ values calculated at or close to 1 and percent recovery between 80 and 120 %. Quality controls included with each kit performed as expected.

### Transfection protocol

We have previously reported on the stably transduced PKA, Epac 1 and Epac 2 knockdown Raw 264.7 cells and shown that there was a marked reduction in the expression of these proteins [19]. Briefly, Raw 264.7 cells (15,000 cells/ml) were plated and 24 h later cells were incubated, in the presence of hexadimethrine bromide (4 μg/ml), with 10^8^ lentiviral transduction particles corresponding to mouse PKA catalytic alpha subunit shRNA (SHCLNV-NM_008854) EPAC1 (RAPGEF3, SHCLNV-NM_144850) or EPAC2 (RAPGEF4, SHCLNV-NM_019688) with puromycin selection marker for another 24 h, to allow transfection. Media was then replaced with αMEM containing puromycin (1 ug/ml), changing the media every 3 days until selected clones formed. These clones were isolated and expanded until confluence. Scrambled shRNA (SHC002V) was used as control. Permanently silenced clones were kept in culture under puromycin selection.

### Statistical analysis

Statistical differences were determined using one-way or two-way analysis of variance (ANOVA) or Student’s *t* test carried out using GraphPad software (v 6.00, GraphPad Software, Inc.) on a PC. The alpha nominal level was set at 0.05 in all cases. A *P* value <0.05 was considered significant.

## Results

### Apremilast and methotrexate independently prevent inflammation in vitro and in the murine air pouch in vivo model of inflammation

Apremilast has previously been shown to inhibit TNF-α production from human rheumatoid synovial cells and this ameliorates arthritis in the experimental model of collagen-induced arthritis [20]. Interestingly, in the air pouch model, an in vivo model that mimics the synovial cavity, we have also demonstrated that the anti-inflammatory actions of MTX, the cornerstone treatment for rheumatoid arthritis, are mediated in large part, by increasing adenosine levels [17], which, via activation of the A_2A_R, increase intracellular cAMP levels [21]. We therefore sought to study apremilast and MTX combined anti-inflammatory actions in both in vivo and in vitro models.

**Fig. 1:**
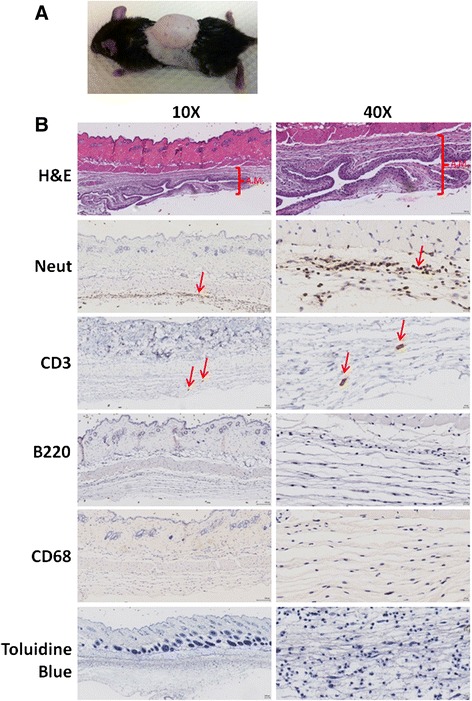
Air pouch model of inflammation. **a** Inflammation in the air pouch was induced as described. **b** H&E reveals the formation of the air pouch membrane (A.M.) and immunohistology staining with specific neutrophil (*Neut*), T cell (CD3), B cell (B220) and macrophage (CD68) markers, and the toluidine blue stain for mast cells was performed

In vivo, an air pouch was formed on the dorsum of the mice (Fig. 1a), and the characteristic air pouch membrane was formed (A.M.; H&E on Fig. 1b) [22] with a cell infiltrate comprised almost exclusively of neutrophils. Among cells forming the cellular infiltrate, we also found a small quantity of CD3^+^ T cells, but we did not detect any B cells, macrophages or mast cells, as analyzed by immunohistochemistry with B220 and CD68 markers, and by toludine blue stain, respectively.

**Fig. 2:**
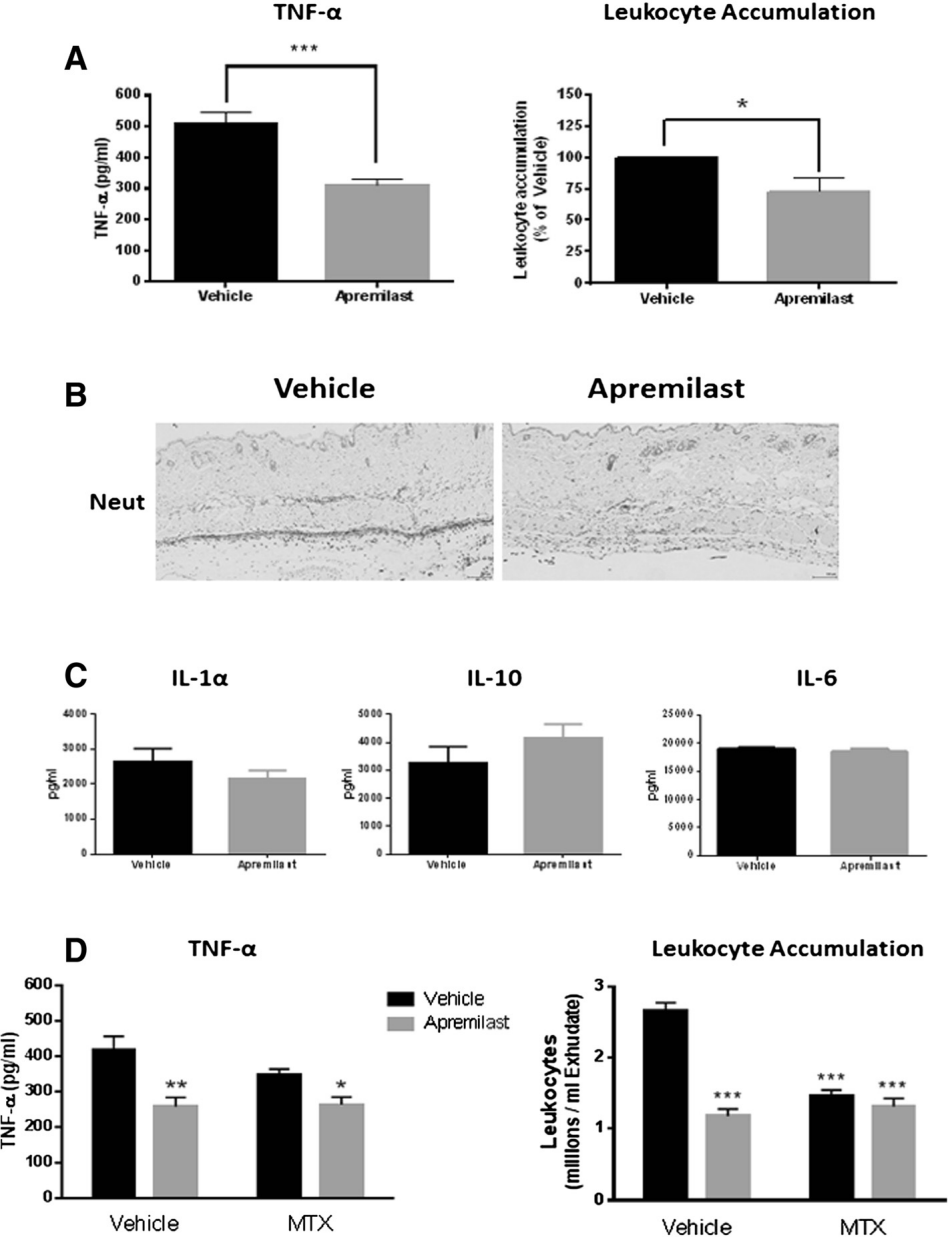
Apremilast and methotrexate (*MTX*) prevent inflammation in the air pouch independently. Mice were orally treated with apremilast (5 mg/Kg), and inflammation in the air pouch was induced as described under “Materials and methods”. **a** TNF-α and leukocyte accumulation were quantified in the exudates of the air pouch. **b** Immunohistology reveals a decrease in the number of neutrophils (*Neut*) by apremilast treatment. **c** IL-1α, IL-6 and IL-10 levels were measured in the air pouch exudates with the Luminex multiplex technology. **d** TNF-α and leukocyte were quantified in the air pouch exudates in mice after weekly intraperitoneal injections of MTX (1 mg/Kg) for 4 weeks prior to apremilast treatment. Data represent mean ± standard error of the mean of at least three independent experiments. Statistical analysis was performed by the Student’s *t* test or one-way ANOVA, where ***P <0.001, **P <0.01 and *P <0.05 vs vehicle

Apremilast, orally administered (5 mg/Kg), significantly inhibited TNF-α production in the air pouch by 39 % (61 ± 6 % of vehicle, *P* <0.001) and diminished (by 28 %) the number of leukocytes present (72 ± 12 % of vehicle, *P* <0.05; Fig. 2a). In agreement, immunohistologic analysis shows that neutrophil accumulation in the air pouch membrane was dramatically reduced by apremilast (Fig. 2b). We measured different mediators of inflammation with the Luminex multiplex platform and found that apremilast treatment did not significantly change the levels of IL-1α, IL-10 and IL-6 in the air pouch exudates (Fig. 2c).

We next pretreated mice with low-dose MTX (1mg/Kg, one dose per week for 4 weeks) prior to apremilast treatment, and studied the inflammation of the air pouch. As shown in Fig. 2d, both apremilast and MTX decreased leukocyte accumulation, and apremilast significantly reduced TNF-α levels. When administered together there was no additive reduction of either TNF-α or leukocyte accumulation in the air pouch. Similarly, no differences were found for the levels of IL-1α, IL-6 and IL-10 treated with apremilast + MTX or apremilast alone (not shown). Because many, but not all of the actions of MTX, working through A_2A_R, were identical and not additive, our results and prior published data suggest that the actions of these agents in suppressing inflammation might be mediated by similar signaling pathways. Thus, we analyzed the intracellular pathways activated by apremilast in vitro.

### Apremilast increases intracellular cAMP and inhibits TNF-α release by LPS in the mouse macrophage Raw 264.7 cell line

**Fig. 3:**
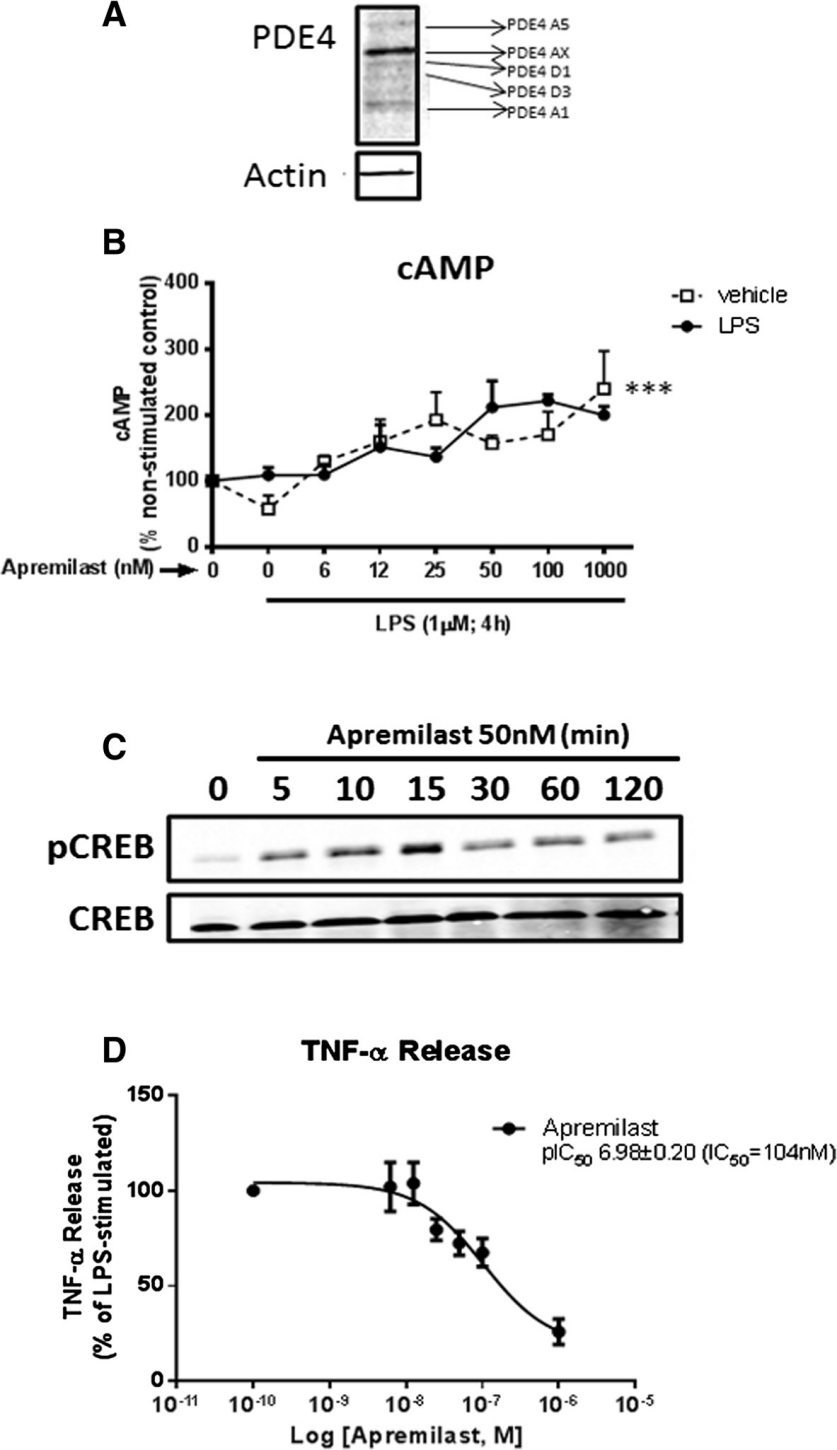
Apremilast inhibits lipopolysaccharide (*LPS*)-induced TNF-α release via cyclic adenosine monophosphalphate (cAMP). **a** Western blot of PDE4 was performed in the Raw 264.7 cell line. **b** Raw 264.7 cells were incubated with increasing concentrations of apremilast 30 minutes before incubation with or without LPS 1 μM during 20 minutes. Intracellular cAMP levels were then measured as described under “Materials and methods”. **c** Western blot of p-cAMP responsive element binding protein (*p-CREB*) and *CREB* shows that apremilast promotes CREB phosphorylation after incubation with LPS 1 μg/ml for 30 minutes. **d** Cumulative concentration response curves to apremilast (6 nM−1 μM) were performed in the Raw 264.7 cells 30 minutes before incubation with LPS 1 μM during 4 h. IC_50_ values were determined as described under “Materials and methods”. Data represent means ± standard error of the mean of four independent experiments. Statistical analysis was performed by two-way ANOVA: apremilast ***P <0.001, LPS: not significant. PDE4 phosphodiesterase 4,

Apremilast inhibits PDE4 with an IC_50_ of 74 nM using 1 μM cAMP as substrate [16]. Although apremilast is not selective for the different PDE4 isoforms (PDE4A4, PDE4B2, PDE4C2 and PDE4D3), as studied with recombinant enzymes, it is indeed PDE4-selective as it did not show significant inhibition of other PDE families at 10 μM [16]. As shown in Fig. 3a, the Raw 264.7 cell line expressed several different isoforms of PDE4 and apremilast significantly increased intracellular cAMP, whether or not cells were challenged by LPS (Fig. 3b, two-way ANOVA analysis; apremilast vs control, *P* <0.001; apremilast plus LPS vs vehicle, not significant), which suggests that the apremilast-mediated cAMP increase is independent of macrophage activation. Consistent with the functional effects of the apremilast-induced increase in cAMP concentrations, apremilast promoted phosphorylation of CREB (Fig. 3c), in agreement with the hypothesis that apremilast activates the anti-inflammatory cAMP/p-CREB pathway [23].

As expected, LPS increased TNF-α release (from 1,345 ± 273 pg/ml to 9,624 ± 1,755 pg/ml; *P* <0.01). A dose−inhibition curve was performed to analyze the impact of increasing concentrations of apremilast showing that apremilast inhibited TNF-α release by LPS with an IC_50_ of 104 nM (pIC_50_ = 6.98 ± 0.2; Fig. 3d), which almost exactly replicates previous reported TNF-α inhibition by apremilast on peripheral blood mononuclear cells (PBMCs) (IC_50_ = 110 nM) and which is similar to the potency of apremilast for PDE4 enzymatic inhibition (IC_50_ = 74 nM) [24]. These results are clearly consistent with the hypothesis that apremilast inhibits TNF-α by increasing intracellular cAMP levels.

### A_2A_R, but not A_2B_R, activation and apremilast exert additive TNF-α inhibition

**Fig. 4:**
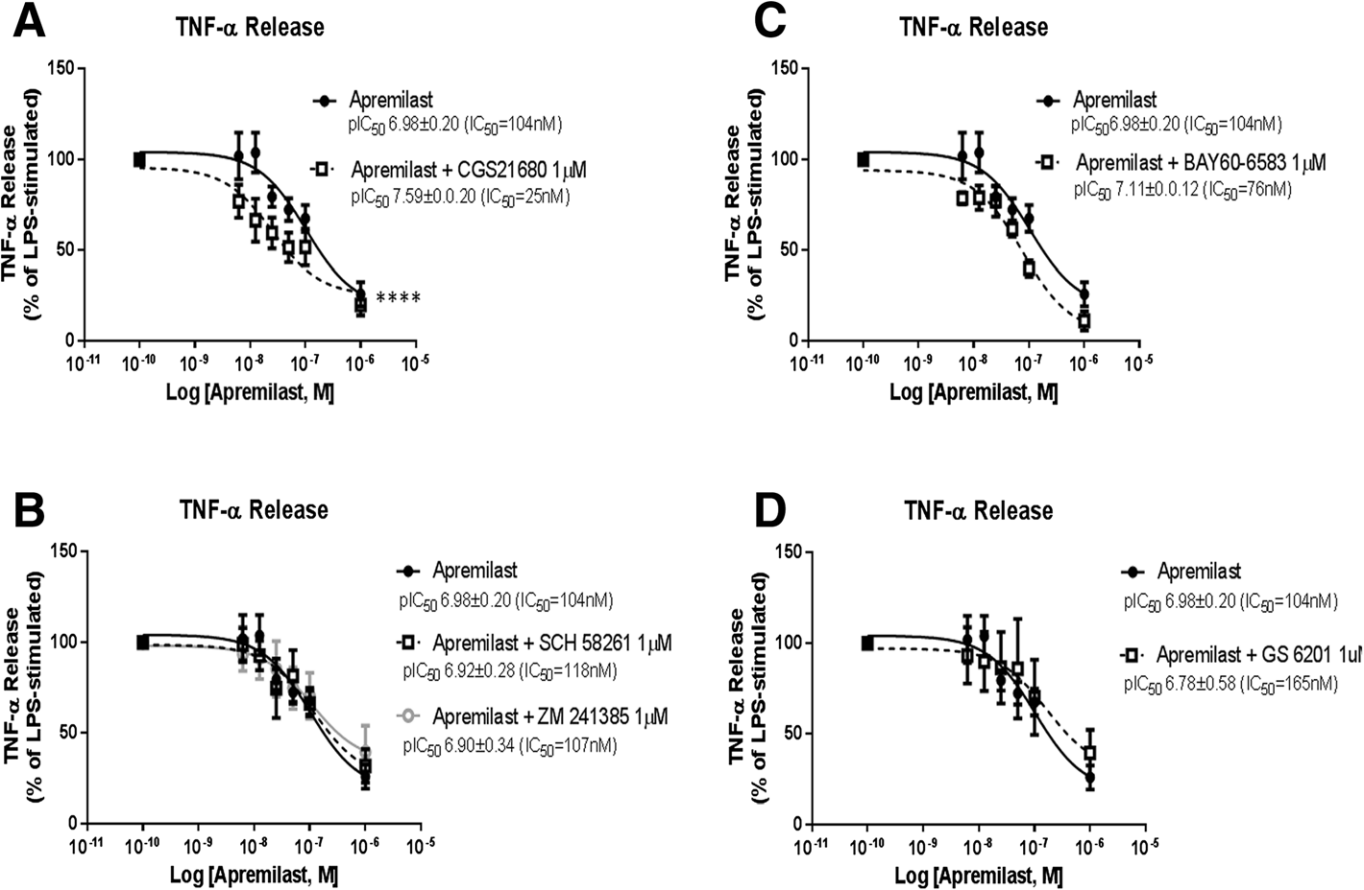
Combined effects adenosine A_2_A receptor (A_2A_R), adenosine A_B_A receptor (A_2B_R) and apremilast on inhibition of TNF-α release. Cumulative concentration response curves to apremilast (6 nM−1 μM) were performed in the Raw 264.7 cells 30 minutes before incubation with lipolysaccharide (*LPS*) 1 μM during 4 h. We added CGS21680 1 μM (**a**), SCH 58261 1 μM or ZM 241385 1 μM (**b**), BAY60-6583 1 μM (**c**) or GS 6201 1 μM (**d**) 15 minutes before apremilast. IC_50_ values were determined as described under “Materials and methods”. Data represent means ± standard error of the mean of three to four independent experiments

Adenosine, a purine nucleoside generated by the dephosphorylation of adenine nucleotides, exerts potent anti-inflammatory actions [21]. Indeed, adenosine inhibits TNF-α, IL-6 and IL-12 release and augments IL-10 production stimulated by LPS mostly through activation of the A_2A_R, a G protein coupled receptor [25–27]. Moreover, there is evidence that the A_2B_R exerts anti-inflammatory actions as well, as it was found that A_2B_R augments LPS-induced IL-10 production in the Raw 264.7 cell line [28]. As both the A2AR and the A2BR couple to Gs protein and increase intracellular cAMP production [21] we hypothesized that apremilast may enhance the actions of both A_2A_R and A_2B_R. When the specific A_2A_R agonist CGS21680 (1 μM) was added to apremilast there was a significant reduction in the IC_50_ for apremilast inhibition of TNF-α release, from 104 nM to 25 nM (pIC_50_ of apremilast + vehicle vs pIC_50_ of apremilast + CGS21680 1 μM: *P* <0.0001, Student’s *t* test; Fig. 4a), indicating that A_2A_R activation and apremilast were additive. However, co-stimulation with CGS21680 and apremilast did not further increase intracellular cAMP levels when compared to either agent alone, though there was some additivity noted at the lower concentrations of CGS21680 and apremilast (Additional file 1: Figure S1). Interestingly, when the impact of two different A_2A_R antagonists (SCH58261 1 μM and ZM241385 1 μM) on the TNF-α inhibition by apremilast was studied, no significant changes were found (Fig. 4b), indicating that A_2A_R blockade alone does not prevent apremilast inhibition of TNF-α despite the interaction between A_2A_R activation and PDE4 inhibition. Further corroboration that diminished A_2A_R activity does not interfere with apremilast, is provided by the finding that when the A_2A_R was knocked down (A_2A_R shRNA: 54 % expression compared to non-target shRNA) apremilast inhibited TNF-α production by LPS to a similar extent (non-target shRNA: 81 ± 2 % TNF-α inhibition vs A_2A_R shRNA: 66 ± 9 %, *P* <0.05).

Next, we studied the impact of a specific A_2B_R agonist (BAY60-6583 1 μM) and a specific A_2_BR antagonist (GS 6201 1 μM) on apremilast-mediated inhibition of TNF-α release. Neither the specific A_2B_R agonist nor the antagonist significantly affected apremilast-mediated inhibition of TNF-α release (Fig. 4c, c), indicating that the A_2B_R does not modulate the anti-inflammatory effects of apremilast.

**Fig. 5:**
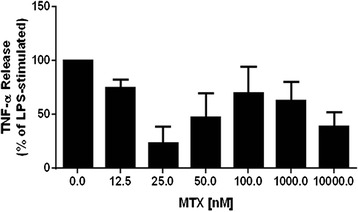
Methotrexate (*MTX*) inhibits TNF-α release upon lipopolysaccharide (*LPS*) challenge. Raw 264.7 cells were incubated with increasing concentrations of MTX 24.0 h and 1.5 h before incubation without LPS 1 μM during 4 h. TNF-α levels were then measured as described under “Materials and methods”. Data represent means ± standard error of the mean of four independent experiments

Previous work has shown that MTX exerts its anti-inflammatory actions by increasing adenosine activation of the A_2A_R [25–27], so we analyzed the impact of MTX on TNF-α release by LPS. As expected from previous reports studying the anti-inflammatory mechanism of MTX in vitro [29], MTX inhibited the TNF-α increase upon LPS challenge by as much as 77 ± 15 % (MTX 25 nM; n = 3) in the Raw 264.7 cell line (Fig. 5). However, the combination of MTX + apremilast at a wide range of concentrations of both agents (apremilast 0.1, 50.0 and 1000.0 nM; MTX 0.12, 0.25, 0.5, 1.0 and 10.0 μM) did not differ from apremilast alone with respect to inhibition of TNF-α (not shown), suggesting that the mechanisms of both MTX and apremilast regulation of TNF-alpha are overlapping.

### Role of PKA and Epac1/2 in the anti-inflammatory actions of apremilast

In eukaryotic cells, the effects of cAMP are mainly mediated by two ubiquitously expressed intracellular cAMP-regulated signaling proteins, PKA and the Exchange protein directly activated by cAMP/cAMP-regulated guanine nucleotide exchange factors (Epac1/2) [30], and by the cyclic nucleotide-gated ion channels [31]. We next examined the role of PKA and Epac1/2 in the anti-inflammatory effects mediated by PDE4 inhibition by apremilast.

**Fig. 6:**
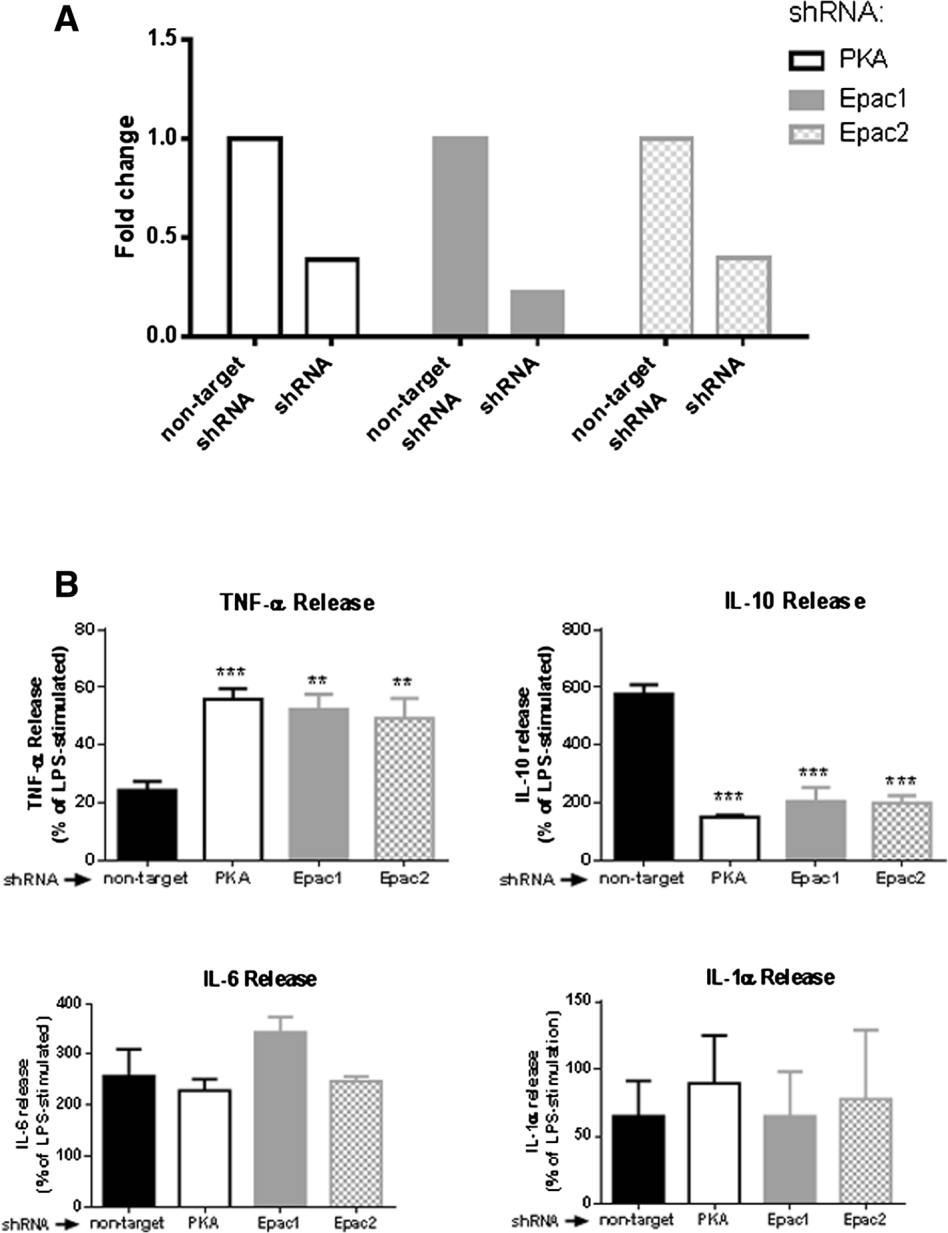
Effect of Protein kinase A (*PKA*), Exchange protein directly activated by cAMP (*Epac*)1 and Epac2 knockdown on the action of apremilast. **a** Transfection of Raw 264.7 cells with shRNA for PKA, Epac1 or Epac2 reduce PKA, Epac1 and Epac2 expression, respectively, as shown by RT-PCR. **b** PKA, Epac1 and Epac2 knockdown Raw 264.7 cells were incubated with apremilast 100nM for 30 minutes before incubation with or without lipopolysaccharide (*LPS*) 1 μM during 4 h. Levels of TNF-α, IL-10, IL-6 and IL-1α were analyzed by ELISA or with the Luminex multiplex technology as described under “Materials and methods”. Data represent means ± standard error of the mean of three independent experiments. Statistical analysis was performed by one-way ANOVA with the Bonferroni posttest correction; ***P <0.001, **P <0.01 vs non-target

We have previously shown that knocking down Epac1, Epac2 or PKA in the RAW264.7 cell line by cellular transduction with lentiviruses that express selective shRNA yields more selective information about the roles of Epac1, Epac2 or PKA in regulating cellular functions than their respective pharmacological inhibitors BFA [19] or the PKA inhibitor TTYADFIASGRTGRRNAIHD [32]. We confirmed that these previously permanently transduced cells expressed less messaging for the target proteins and observed a specific decrease of 61 %, 77 % and 60 %, respectively, for the PKA, Epac1 and Epac2 knockdown cells (Fig. 6a). In agreement, in the Raw 264.7 cell line, protein levels were dramatically decreased with the specific shRNAs (previously reported in [19] and in Additional file 2: Figure S2). In non-target shRNA-transfected cells, LPS increased TNF-α from 442.6 ± 57.2 to 9731.6 ± 2500.4 pg/ml (*P* <0.05) and apremilast treatment (100 nM) inhibited TNF-α production by 76 ± 3 % (Fig. 6b), whereas knockdown of PKA, Epac1 and Epac2 reduced apremilast-mediated inhibition to 44 ± 4%, 48 ± 5 % and 51 ± 7 % inhibition, respectively. These results suggest that cAMP-mediated inhibition of TNF-α production is mediated via PKA and Epac1/2 activation.

To further study the anti-inflammatory effects of apremilast we analyzed the effect of apremilast on cytokine release with the Luminex multiplex platform. Among cytokines increased by LPS (IL-10, IL-6 and IL-1α), in non-target shRNA transfected cells apremilast further increased IL-10 (from 32 ± 7 to 181 ± 33 pg/ml, *P* <0.05). Apremilast showed a trend to increase IL-6 in this murine cell line (from 811 ± 416 to 1,986 ± 881 pg/ml, *P* <0.05) and modestly diminished the LPS-mediated increase of IL-1α (from 149 ± 26 to 97 ± 35 pg/ml, *P* <0.05). Interestingly, all three knockdowns for PKA, Epac1 and Epac2, prevented the apremilast-mediated increase of IL-10, but knockdown of these signaling molecules did not affect apremilast-mediated regulation of IL-6 and IL-1α levels (Fig. 6b, lower panels).

## Discussion

PDE4 is the predominant cAMP-selective phosphodiesterase regulating the function of inflammatory cells [5] and selective PDE4 inhibitors have therefore generated great interest for the treatment of immune diseases, including inflammatory arthritis. Unfortunately most PDE4 inhibitors, such as rolipram, induce a variety of side effects, including nausea and emesis, because PDE4 is also highly expressed in the central nervous system [10]. As a consequence, early clinical studies with PDE4 inhibitors failed to demonstrate benefit due to the high incidence of these adverse effects [11].

Apremilast was discovered through evaluation of substitutions on a chemical scaffold, to optimize the structure-activity relationship of a particular series of PDE4 inhibitors [14, 15]. The orally available PDE4 inhibitor inhibits spontaneous TNF-α production from human synovial membrane cultures and reduces the severity of disease in murine models of arthritis with similar efficacy to rolipram, but without any significant adverse effects [20]. Moreover, apremilast has been well-tolerated in clinical trials, with a favorable benefit to risk profile [33, 34] and has recently been approved for the therapy of psoriatic arthritis.

In the air pouch model of inflammation in vivo, which mimics the synovial cavity in rheumatoid arthritis, we found an inflammatory exudate composed primarily of neutrophils with a small number of T cells (Fig. 1). Apremilast dramatically decreased the accumulation of neutrophils and inhibited TNF-α and IL-1α production, while at the same time increasing the levels of the anti-inflammatory cytokine IL-10 (Fig. 2), further corroborating the pleiotropic anti-inflammatory actions of apremilast.

Apremilast is a well-described inhibitor of PDE4 [14], as measured on PDE4 and recombinant PDE4 isolated from cells [14, 16, 35]. The present work shows that apremilast increases intracellular cAMP in cell systems, consistent with PDE4 inhibition and previous data [15]. We found that apremilast promoted a significant increase of cytosolic cAMP in the Raw 264.7 cell line independently of LPS stimulation (Fig. 3b). Moreover, the potency of TNF-α inhibition by apremilast in these cells (IC_50_ 104 nM) was nearly identical to that for its reported inhibition of PDE4 activity (IC_50_ 74nM) and TNF-α inhibition in peripheral blood mononuclear cells (IC_50_ = 110 nM, [16]). As expected, these findings indicate that PDE4 inhibition by apremilast suppresses TNF-α production by increasing cAMP.

Interestingly, the anti-inflammatory action of MTX, the cornerstone drug for the treatment of rheumatoid arthritis and other inflammatory diseases [36], depends on the extracellular conversion of adenine nucleotides to adenosine [17]. Adenosine was described as an anti-inflammatory agent nearly 30 years ago [21]. By binding the A_2A_R, which signals almost exclusively by increasing intracellular cAMP levels [37], adenosine has been shown to inhibit TNF-α, IL-6 and IL-12 release, while it augments IL-10 production induced by LPS [25–27]. We hypothesized that apremilast and A_2A_R activation could interact at the cAMP level. Therefore, we investigated the impact of stimulation or blockade of the A_2A_R on the apremilast-mediated inhibition of TNF-α production, finding that the A_2A_R-specific agonist CGS21680 increases the potency of apremilast from an IC_50_ of 104 nM to 25 nM (Fig. 4a). However, in the presence of apremilast, CGS21680 did not further increase the levels of cAMP (Additional file 1: Figure S1) and A_2A_R pharmacological blockade (Fig. 4b) did not further alter apremilast inhibition of TNF-α. This apparent discrepancy might be explained by an insufficient amount of endogenous adenosine production in the cellular system. Similarly, despite its potential link to enhanced cAMP levels, A_2B_R activation or blockade did not affect TNF-α inhibition by apremilast (Fig. 4c, d). Moreover, we were surprised to find that apremilast showed a trend to increase IL-6 production in vitro, although we did not detect any increase in vivo. In contrast, stimulation of A_2A_R diminishes IL-6 production [21]. Interestingly, treatment with MTX diminishes circulating IL-6 levels in patients [38, 39] although the effect of the drug alone on IL-6 production in vitro is equivocal [40–42]. In patients with psoriatic arthritis, apremilast has been shown to reduce plasma levels of TNF- α, IL-6, and other pro-inflammatory cytokines and chemokines [43].

The A_2A_R is the only Gs-coupled adenosine receptor subtype that has not been reported to also couple to the Gq-protein [37] and thus, it signals almost exclusively by increasing cAMP levels. The findings reported here suggest that apremilast and A_2A_R activation most likely interact by either transient cAMP signals and/or by compartmentalized cAMP increases, as previously found for prostaglandin E1 (PGE1), which triggers transient increases in the cAMP concentration near the plasma membrane, but not in total intracellular cAMP levels [44]. Indeed, recent studies indicate that there are distinct cAMP-signaling microdomains controlled by specific PDEs, which can differentially regulate independent processes controlled by cAMP [45–47], so this same signaling molecule can have opposing effects within the same cell due to a compartmentalized mechanism. On the other hand, although MTX prevents TNF-α release in vitro (Fig. 5) and inflammation in the air pouch via adenosine release [17], we did not detect additive effects of apremilast + MTX either in vitro (Fig. 5) or in vivo (Fig. 2d), which is in agreement with the finding that no additional benefit or risk is associated with combination of apremilast and methotrexate therapy [48]. Indeed, in a phase 3 study of apremilast demonstrating its efficacy and safety in patients with psoriatic arthritis, the majority of patients included in the trial were on concomitant MTX therapy at baseline [49]. In order to rule out interactions between apremilast and MTX, we tested a wide range of concentrations of both agents in a variety of combinations (apremilast 0.1, 50.0 and 1000.0 nM; MTX 0.12, 0.25, 0.5, 1.0 and 10.0 μM), finding a lack of interaction (data not shown), which may result from the variability of enhanced adenosine release in vitro leading to greater variability in the inhibition due to MTX.

Targeting PDE4 has enormous clinical potential due to its mechanism of action, which leads to increased intracellular cAMP levels in many different inflammatory cells, decreasing T cell and monocyte-derived cytokines such as TNF-α [6–8]. From studies using the PDE4 inhibitors roflumilast and rolipram, it is known that PDE4 inhibition leads to decreased TNF-α gene expression by a cAMP, PKA and NF-κB-dependent mechanism [9, 24]. Similarly, increased expression of the anti-inflammatory cytokine IL-10 is enhanced by PDE4 inhibitors in a PKA-dependent manner [50]. However, the discovery of the exchange protein directly activated by cAMP (Epac) suggested that the cAMP-mediated signaling mechanism is much more complex [30], so that cAMP exerts its effects not only via PKA activation, but also via Epac [51]. In particular, it has been suggested that many anti-inflammatory effects of cAMP are mediated via the PKA pathway, while there are no major anti-inflammatory actions attributed to Epac [52, 53]. Moreover, prior studies have yielded results that support the suggestion that Epac activation following PDE4 inhibitor-mediated cAMP increases has pro-inflammatory effects and that addition of an Epac inhibitor to the PDE4 inhibitor treatment may lead to greater anti-inflammatory efficacy [53]. Although our studies demonstrate that the effects of apremilast on TNFα secretion are mediated by both PKA and Epac1/2 activation (Fig. 6b) it is likely that the different inflammatory stimuli used in the prior and current studies may account for this apparent discrepancy, LPS stimulation (present work) vs no stimulus ([53]).

These studies were carried out using a murine model of synovial inflammation, the air pouch model, and using murine macrophage cell lines. Both of these model systems reflect inflammatory events as they occur in patients, although neither of them are a perfect model. In the air pouch model inflammation is induced by the injection of carrageenan into an artificially created pouch that resembles the synovium, but the lining cells, while likely mesothelial in origin, are not synoviocytes and the inflammatory stimulus is not physiological. Similarly, the Raw 264.7 cells used here are derived from murine macrophages but there are likely other mutations in these cells that have rendered them immortal and which may exert some effect on the response to drugs or inflammatory stimuli. Nonetheless, these model systems permit a detailed study of the inflammatory milieu and support molecular probes of inflammatory signaling that are not possible in inflamed human synovium or primary human cells.

## Conclusions

In summary, we report here that the novel PDE4 inhibitor apremilast is a potent inhibitor of inflammation via cAMP, PKA, and Epac1/2, and that interactions with the A2AR are likely due to compartmentalization of cAMP, rather than total cAMP changes. In an in vivo model that mimics the synovial cavity, apremilast exerts a potent anti-inflammatory action, which was not affected by MTX in this model. These results may help to explain the cAMP-dependent mechanism of action of apremilast as currently labeled for use in the treatment of psoriatic arthritis.

## Additional files

Additional file 1: Figure S1. Adenosine A2A receptor (A_2A_R) activation and apremilast do not additively increase responsive element binding protein (*cAMP*). Raw 264.7 cells were incubated with cumulative concentrations of apremilast (6 nM to 1 μM), apremilast + CGS21680 1μM 15 minutes before apremilast, or cumulative concentrations of CGS21680 alone (6 nM to 1 μM), followed by treatment with lipopolysaccharide (*LPS*) 1 μM for 20 minutes. Then, intracellular cAMP levels were measured as described under “Materials and methods”. Data represent means ± standard error of the mean of at least three independent experiments. (TIFF 170 kb) Additional file 2: Figure S2. Permanent transduction of shRNA for protein kinase (*PKA*) dramatically decreases PKA protein expression in the Raw 264.7 cell line, as determined by western blotting. (TIFF 268 kb)

## Abbreviations

A_2A_R: adenosine A2A receptor
A_2B_R: adenosine A2B receptor
ANOVA: analysis of variance
BCA: bicinchoninic acid
cAMP: cyclic adenosine monophosphalphate
CREB: cAMP responsive element binding protein
ELISA: enzyme-linked immunosorbent assay
EPAC1/2: Exchange protein directly activated by cAMP
H&E: hematoxylin and eosin
IL: interleukin
LPS: lipopolysaccharide
MTX: methtrexate
PBS: phosphate-buffered saline
PDE4: phosphodiesterase 4
PKA: protein kinase A
shRNA: short hairpin RNA
TNFα: tumor necrosis factor alpha
