# Supplementary figures and images for: Apremilast, a novel phosphodiesterase 4 (PDE4) inhibitor, regulates inflammation through multiple cAMP downstream effectors
Source: Arthritis Res Ther. 2015 Sep 15;17(1):249. doi: 10.1186/s13075-015-0771-6 (PMC4570588; doi:10.1186/s13075-015-0771-6)

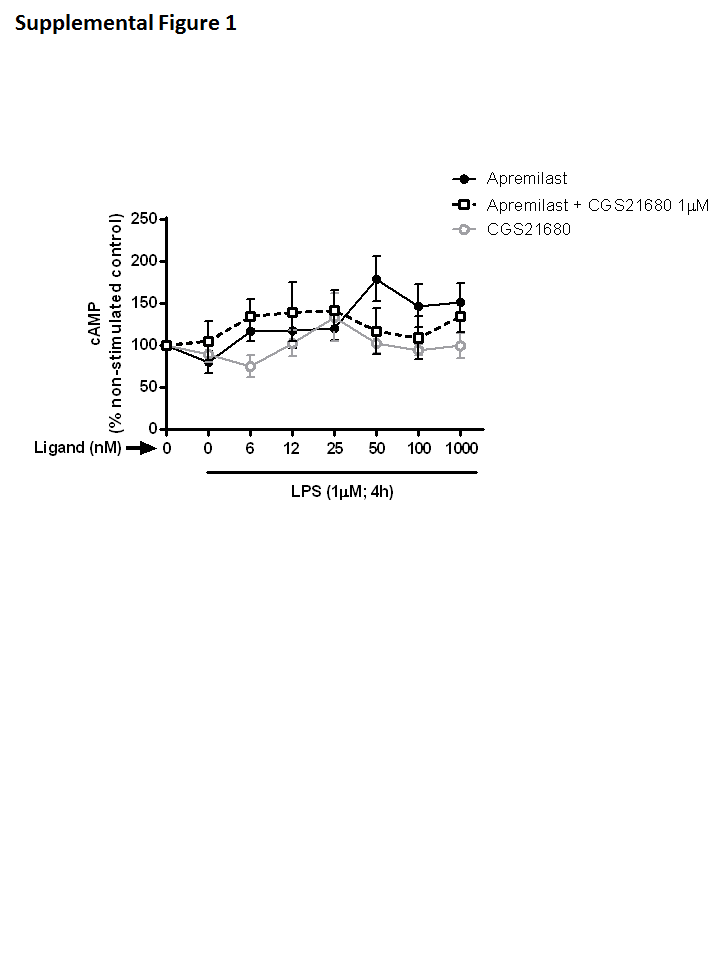

Supplement: Additional file 1: Figure S1. — Adenosine A2A receptor (A2AR) activation and apremilast do not additively increase responsive element binding protein (cAMP). Raw 264.7 cells were incubated with cumulative concentrations of apremilast (6 nM to 1 μM), apremilast + CGS21680 1μM 15 minutes before apremilast, or cumulative concentrations of CGS21680 alone (6 nM to 1 μM), followed by treatment with lipopolysaccharide (LPS) 1 μM for 20 minutes. Then, intracellular cAMP levels were measured as described under “Materials and methods”. Data represent means ± standard error of the mean of at least three independent experiments. (TIFF 170 kb) [file 13075_2015_771_MOESM1_ESM.tiff]

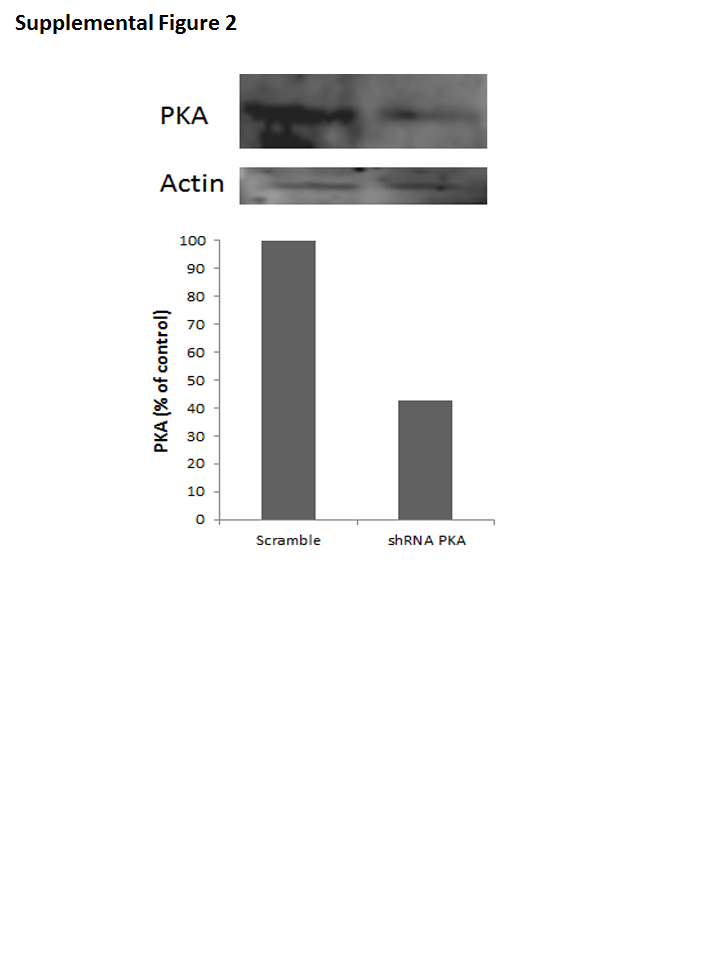

Supplement: Additional file 2: Figure S2. — Permanent transduction of shRNA for protein kinase (PKA) dramatically decreases PKA protein expression in the Raw 264.7 cell line, as determined by western blotting. (TIFF 268 kb) [file 13075_2015_771_MOESM2_ESM.tiff]
